# Privacy-preserving Quantum Sealed-bid Auction Based on Grover’s Search Algorithm

**DOI:** 10.1038/s41598-019-44030-8

**Published:** 2019-05-20

**Authors:** Run-hua Shi, Mingwu Zhang

**Affiliations:** 1 School of Computer Science, Hubei University of Technology, Wuhan City, 430068 China; 2 School of Control and Computer Engineering, North China Electric Power University, Beijing City, 102206 China

**Keywords:** Information technology, Quantum information

## Abstract

Sealed-bid auction is an important tool in modern economic especially concerned with networks. However, the bidders still lack the privacy protection in previously proposed sealed-bid auction schemes. In this paper, we focus on how to further protect the privacy of the bidders, especially the non-winning bidders. We first give a new privacy-preserving model of sealed-bid auction and then present a quantum sealed-bid auction scheme with stronger privacy protection. Our proposed scheme takes a general state in N-dimensional Hilbert space as the message carrier, in which each bidder privately marks his bid in an anonymous way, and further utilizes Grover’s search algorithm to find the current highest bid. By *O*(*lnn*) iterations, it can get the highest bid finally. Compared with any classical scheme in theory, our proposed quantum scheme gets the lower communication complexity.

## Introduction

Nowadays, quantum computations and quantum communications^1^ have received extensive attention and gained lots of promising achievements, e.g., quantum cryptography^2^, quantum teleportation^3^ and quantum artificial intelligence^4,5^.

Early 70s in the last century, Stephen Wiesner first presented the idea of quantum cryptography (e.g., quantum money). However, unfortunately, his innovative idea could not be immediately accepted at that time. Until 1984, C. H. Bennett and G. Brassard^6^ revived the research of quantum cryptography by presenting famous quantum key distribution (QKD) protocol, later called BB84 protocol.

The security of quantum cryptography is guaranteed by the physical principles of quantum mechanics, so it can provide unconditional security in theory. Since Bennett and Brassard presented the first quantum key distribution (i.e., BB84 QKD) protocol, quantum cryptography has been widely studied and rapidly developed. Nowadays, many results have been reported, such as quantum secret sharing^7^, quantum secure direct communication^8–10^, quantum encryption^11^, quantum signature^12–14^, quantum authentication^15,16^, and blind quantum computation^17,18^.

In addition, there are also many well-known issues involving the protection of privacy in classical setting such as electronic voting, electronic auction, electronic payment, and so on. Furthermore, these issues have also been studied extensively in quantum setting, and accordingly there have appeared the corresponding quantum protocols, such as quantum voting^19^, quantum auction^20^, quantum e-payment^21^, and so on.

In this paper, we focus on quantum auction, especially a specific type of quantum auction, i.e., quantum sealed-bid auction (QSA). In currently existing QSA schemes, there is only one winning bidder, who will win the auction finally, but the auctioneer needs to know all bids of all bidders, including the non-winning bidders. That is, even if the non-winning bidder cannot win the auction, he still needs to privately send his bid to the auctioneer. In certain settings, these QSA schemes do not meet the higher secure requirements, because the non-winning bidders lack the privacy protection, which has been the focus of everyone’s attention in modern society. In this paper, we mainly consider how to further protect the privacy of the non-winning bidders in QSA.

## Related Works

Electronic auction plays an important role in modern economy especially concerned with networks. Generally, electronic auction can be mainly classified into three categories: English auction, Dutch auction and Sealed-bid auction. The traditional English auction is a public ascending price auction. In this auction, the auctioneer first gives a base price, and then some bidder bids a higher price than the base price. Furthermore, the next bidder outbids the last bidder, and the process continues until no one else bids a higher price. Finally, the item is sold to the highest bidder at the highest bid. On the contrary, the Dutch auction is a public descending price auction. The auctioneer in Dutch auction begins with a high asking price which is lowered until some bidder is willing to accept the auctioneer’s price. Difference from the former two auctions, the sealed-bid auction needs to protect the privacy of the bids and ensure the fairness among the bidders. That is, any eavesdropper cannot get any private information about the bids, and the auctioneer cannot help any bidder to win the auction unfairly. During traditional sealed-bid auction, the bidder does not know the bids of others. After all bids are transmitted privately to the auctioneer, the auctioneer selects out the highest bid and announces it and the corresponding winner.

The first quantum sealed-bid auction protocol was proposed by Naseri in 2009^20^. The auction protocol introduced a multi-party quantum secure direct communication protocol to privately transmit the bids. However, Qin *et al*.^22^ and Yang *et al*.^23^ independently pointed out that there was a secure flaw in Naseri’ protocol, i.e., a malicious bidder could obtain all private bids without being found by performing double Controlled NOT attack or using fake entangled particles. Then they improved Naseri’s original protocol by inserting some decoy particles into the transmitted particles. In addition to the detecting strategy of the decoy particles, there still appeared other defense strategies^24,25^ to prevent these attacks. Furthermore, Zhao *et al*.^26^ found that these previously proposed protocols were unfair, i.e., a malicious bidder could collude the dishonest auctioneer to perform a collusion attack to win the auction unfairly. Accordingly, they presented a security protocol for QSA with post-confirmation^26^. Subsequently, in order to enhance the security of QSA or ensure the feasibility of QSA, many quantum protocols with post-confirmation were proposed^27–33^. In 2017, we presented an economic and feasible quantum sealed-bid auction protocol based on single photons in both the polarization and the spatial-mode degrees of freedom^34^. In our protocol, the post-confirmation mechanism uses single photons instead of entangled EPR pairs, and it does not require quantum memory. Therefore, our protocol is a practical and feasible quantum sealed-bid auction.

In all previously proposed quantum sealed-bid auction (QSA) protocols, it requires all bidders to send their real bids to the auctioneer. Even if the bidder can not win the auction, the auctioneer also knows his or her real bid. However, in practical settings, the bidders who will not be able to win the auction don’t want to reveal their real bids. That is, the non-winning bidders lack the privacy protection in current QSA schemes. In this paper, we present a strong privacy-preserving QSA model. In our model, anyone cannot get the real bid of other bidders, even for the auctioneer. So the privacy of the bidders can be better protected in our model. In addition, the bids of the bidders are anonymous, i.e., no one can discern who these bids belong to. Furthermore, we design a novel privacy-preserving QSA scheme based on Grover’s search algorithm. The proposed scheme not only guarantees the correctness and fairness of the auction, but also ensures the privacy and anonymity of the bidders, even for the auctioneer. Compared with the current existing quantum sealed-bid auction, our proposed scheme can provide stronger privacy protections, which are urgently requirements in modern network society.

## Results and Discussion

### Privacy-preserving quantum sealed-bid auction

#### System model

**Figure 1:**
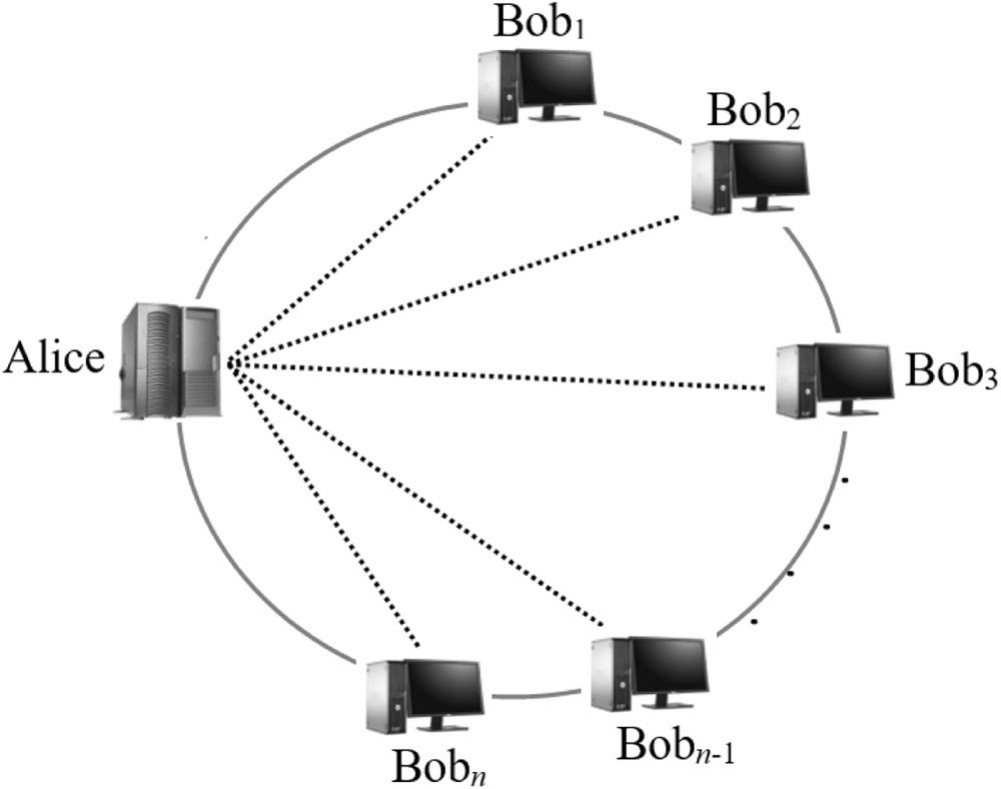
A system model of QAS.

Here we first present our system model for privacy-preserving quantum sealed-bid auction (PQSA), in which there are two kinds of participants, i.e., an auctioneer (Alice) who wants to sell an item at the highest possible price and *n* bidders (*Bob*_1_, *Bob*_2_, …, *Bob*_*n*_) who want to buy the item alone at the lowest possible price. In our PQAS model, suppose that there is a circle quantum channel among the auctioneer and all bidders (see the solid line in Fig. 1) and there is a classical channel between any two participants (see the dashed line in Fig. 1).

Initially, Alice has a valuation price (*x*) of the item, and each bidder (*Bob*_*i*_) has a private bid (*x*_*i*_) for the item. Furthermore, we assume that the valuation price and all bids are not changed during the whole auction. Finally, Alice can select out the highest bid. If the highest bid is greater than or equal to her initial valuation price, then she will announce the winner and the highest bid. Otherwise, she will declare the failure to all bidders. In addition, our PQSA should meet the following secure and privacy requirements:

**The auctioneer’s privacy**: All bidders can not get any private information about the auctioneer’s initial valuation price (*x*) before announcing the winner or the failure of the auction.

**The bidder’s privacy**: No one can get the private bid of the bidder without risking the auctioneer’s detection.

**Anonymity**: The bidder’s bid is anonymous for all participants, including the auctioneer. That is, even if a dishonest participant or an outsider attacker gets a bid, he or she cannot identify whose bid it is.

**Public verifiability**: When the winner is announced, anyone can verify the authenticity of the winning bid. This attribute can defend the collusion attack between the malicious bidder and the dishonest auctioneer.

**Fairness**: The auctioneer cannot help a malicious bidder to win the auction illegally without being found by other bidders.

#### Proposed scheme

In the following scheme, we mainly consider the honest-but-curious model, which is similar to the semi-honesty model in the classical setting. That is, the parties honestly execute the protocol, but they try to find out as much as possible about the other inputs despite following the protocol. Furthermore, suppose that the initial valuation price and all bids lie in *Z*_*N*_ = {0, 1, 2, …, *N* − 1}. For simplicity, we assume that all bids are distinct. In addition, we assume that there is a public hash *H*(·).

**Step 1**. Each bidder *Bob*_*j*_ (*j* = 1, 2, …, *n*) randomly selects an integer *r*_*j*_ ∈ *Z*_*N*_ and computes ${b}_{j}=H({r}_{j}\oplus H({r}_{j}\oplus {x}_{j}))$. Then the bidder *Bob*_*j*_ sends *b*_*j*_ to all other participants by the classical channel. That is, the bidder *Bob*_*j*_ commits *x*_*j*_ to all other participants, but no participant can get *x*_*j*_ only from *b*_*j*_ without *r*_*j*_. In addition, the auctioneer Alice also needs to commit *x* to all bidders, i.e., she selects a random number *r* ∈ *Z*_*N*_, computes $b=H(r\oplus H(r\oplus x))$ and sends *b* to all bidders by the classical channel.

**Step 2**. Repeat the following procedures *p* + *q* times, including the normal procedure (to find the highest bid) *p* times and the test procedure (to detect the dishonesty or attacks) *q* times, where *p* = ln*n*, and *q* is a secure parameter, e.g., *q* = *p*. That is, Alice randomly selects to execute the following normal procedure with the probability of $\frac{p}{p+q}$ or the following test procedure with the probability of $\frac{q}{p+q}$.

**Figure 2:**
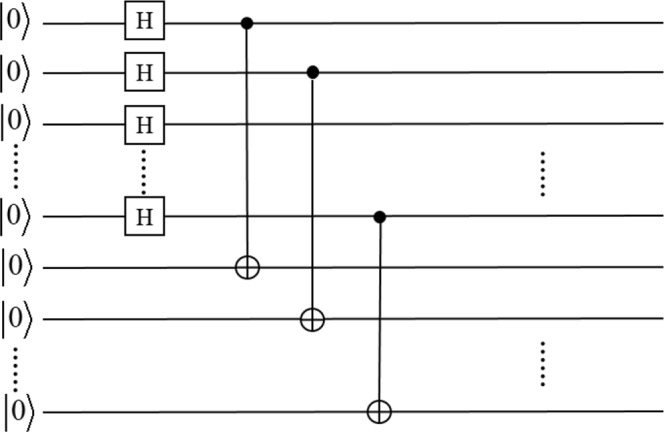
Quantum circuit for the preparation of the initial state.

The normal procedure: (1.1) Alice first prepares a general state ${|\psi }_{h}=\frac{1}{\sqrt{N}}\sum _{i=0}^{N-1}|i{\rangle }_{h}$ and a basis state |0〉_*t*_, which are both log*N* qubits. Furthermore, Alice performs log*N* CNOT gate operators^35^ on the product state $|\psi {\rangle }_{h}|0{\rangle }_{{\rm{t}}}$, where each qubit of the first log*N* qubits is the control qubit and the corresponding qubit of the second log*N* qubits is the target qubit (see Fig. 2). Here we call the resultant state |*ψ*_0_〉, which is written as

1 $$\begin{array}{ccc}|{\psi }_{0}\rangle  & = & CNO{T}^{\otimes {\rm{l}}{\rm{o}}{\rm{g}}N}|\psi {\rangle }_{h}|0{\rangle }_{t}\\  & = & CNOT(1,\,{\rm{l}}{\rm{o}}{\rm{g}}\,N+1)\otimes CNOT(2,\,{\rm{l}}{\rm{o}}{\rm{g}}\,N+2)\ldots \\  &  & \otimes CNOT({\rm{l}}{\rm{o}}{\rm{g}}\,N,2\,{\rm{l}}{\rm{o}}{\rm{g}}\,N)(\frac{1}{\sqrt{N}}\sum _{i+0}^{N-1}{|i\rangle }_{h}|0{\rangle }_{t})\\  & = & \frac{1}{\sqrt{N}}\sum _{i=0}^{N-1}{|i\rangle }_{h}|i{\rangle }_{t.}\end{array}$$

Clearly, |*ψ*_0_〉 is an entangled state. Here, the subscript *h* and *t* denote two registers, where the register *h* will stay at home and the register *t* will be transmitted through the quantum channel. Then Alice sends the register *t* to the first bidder *Bob*_1_ through the quantum channel.

(1.2) After receiving the register *t*, the bidder *Bob*_1_ prepares a basis state |0〉 in an auxiliary register, and applies an oracle operator ${U}_{Bo{b}_{1}}$ to the register *t* and the auxiliary register, where the oracle operator ${U}_{Bo{b}_{1}}$ is defined by

2 $${U}_{Bo{b}_{1}}:\frac{1}{\sqrt{N}}{\sum }_{i=0}^{N-1}|i{\rangle }_{t}\otimes |0\rangle \to \frac{1}{\sqrt{N}}{\sum }_{i=0}^{N-1}|i{\rangle }_{t}|0\oplus f(i,{x}_{1})\rangle ,$$

with

3 $$f(i,{x}_{1})=\{\begin{array}{ll}1 & if\,i={x}_{1}\\ 0 & else\end{array}.$$

Let $$|{\psi }_{1}=\frac{1}{\sqrt{N}}{\sum }_{i=0}^{N-1}|i{\rangle }_{h}|i{\rangle }_{t}|f(i,{x}_{1})\rangle $$ (i.e., the state of the whole quantum system). Obviously, $$|{\psi }_{1}=\frac{1}{\sqrt{N}}[|{x}_{1}{\rangle }_{h}|{x}_{1}{\rangle }_{t}|1\rangle +$$$$\sum _{i\ne {x}_{1}}|i{\rangle }_{h}|i{\rangle }_{t}|0\rangle ]$$. That is, the oracle operator $${U}_{Bo{b}_{1}}$$ is utilized to mark the item *x*_1_.

(1.3) Furthermore, the bidder *Bob*_1_ sends the two registers (i.e., $\frac{1}{\sqrt{N}}{\sum }_{i=0}^{N-1}|i{\rangle }_{t}|f(i,{x}_{1})\rangle$) to the second bidder *Bob*_2_ through the quantum channel.

(1.4) After receiving $\frac{1}{\sqrt{N}}{\sum }_{i=0}^{N-1}|i{\rangle }_{t}|f(i,{x}_{1})\rangle$, similarly, the bidder *Bob*_2_ applies an oracle operator ${U}_{Bo{b}_{2}}$ to $\frac{1}{\sqrt{N}}{\sum }_{i=0}^{N-1}|i{\rangle }_{t}|f(i,{x}_{1})\rangle$, where the oracle operator ${U}_{Bo{b}_{2}}$ is defined by his bid *x*_2_ as follows:

4 $${U}_{Bo{b}_{2}}:\frac{1}{\sqrt{N}}{\sum }_{i=0}^{N-1}|i{\rangle }_{t}|f(i,{x}_{1})\rangle \to \frac{1}{\sqrt{N}}{\sum }_{i=0}^{N-1}|i{\rangle }_{t}|f(i,{x}_{1})\oplus f(i,{x}_{2})\rangle ,$$

with

5 $$f(i,{x}_{2})=\{\begin{array}{ll}1 & if\,i={x}_{2}\\ 0 & else\end{array}.$$

Let $|{\psi }_{2}\rangle =\frac{1}{\sqrt{N}}{\sum }_{i=0}^{N-1}|i{\rangle }_{h}|i{\rangle }_{t}|f(i,{x}_{1})\oplus f(i,{x}_{2})\rangle$. Furthermore, the bidder *Bob*_2_ sends two transmitted registers (i.e.,$\,\frac{1}{\sqrt{N}}{\sum }_{i=0}^{N-1}|i{\rangle }_{t}|f(i,{x}_{1})\oplus f(i,{x}_{2})\rangle$) to the next bidder *Bob*_3_ though the quantum channel. Afterward, the bidder *Bob*_3_ executes the similar process of the bidder *Bob*_2_, and so on. This process is repeated *n* times in total, so that every bidder has marked his bid by an oracle operator. Then, the final quantum state will be in

6 $$\begin{array}{rcl}|{\psi }_{n}\rangle  & = & \frac{1}{\sqrt{N}}\sum _{i=0}^{N-1}|i{\rangle }_{h}|i{\rangle }_{t}|f(i,{x}_{1})\oplus f(i,{x}_{2})\oplus \cdot \,\cdot \,\cdot \oplus f(i,{x}_{n})\rangle \\  & = & \frac{1}{\sqrt{N}}[{\sum }_{i\notin \{{x}_{1},{x}_{2},\ldots {x}_{n}\}}|i{\rangle }_{h}|i{\rangle }_{t}|0\rangle +{\sum }_{j\in \{{x}_{1},{x}_{2},\ldots {x}_{n}\}}|j{\rangle }_{h}|j{\rangle }_{t}|1\rangle ]\end{array}.$$

(1.5) Finally, the bidder *Bob*_*n*_ sends all remaining qubits of the marked state |*ψ*_*n*_〉 back to the auctioneer Alice through the quantum channel.

(1.6) After receiving the whole state |*ψ*_*n*_〉, Alice again applies ${{\rm{CNOT}}}^{\otimes \mathrm{log}N}$ on two registers *h* and *t*, i.e., the first 2log*N* qubits of |*ψ*_*n*_〉, where each qubit of the first log*N* qubits is the control qubit and the corresponding qubit of the second log*N* qubits is the target qubit. Call the resultant state $|\mathop{\psi }\limits^{ \sim }{\rangle }_{n}$. That is,

7 $$\begin{array}{rcl}|\tilde{\psi }{\rangle }_{n} & = & CNO{T}^{\otimes {\rm{logN}}}|{\psi }_{n}\rangle \\  & = & CNO{T}^{\otimes \mathrm{log}N}{[\frac{1}{\sqrt{N}}\sum _{i=0}^{N-1}|i\rangle }_{h}|i{\rangle }_{t}|f(i,{x}_{1})\oplus f(i,{x}_{2})\oplus \cdot \,\cdot \,\cdot \oplus f(i,{x}_{n})\rangle ]\\  & = & \frac{1}{\sqrt{N}}{\sum }_{i=0}^{N-1}|i{\rangle }_{h}|0{\rangle }_{t}|f(i,{x}_{1})\oplus f(i,{x}_{2})\oplus \cdot \,\cdot \,\cdot \oplus f(i,{x}_{n})\rangle .\end{array}$$

(1.7) Furthermore, Alice measures the second register *t*, i.e., the second log*N* qubits of the whole quantum system, in the computational basis. If the measured result is |0〉, then she will continue to execute the next step; Otherwise she will believe that there is at least one dishonest bidder or outsider attacker and end this auction.

(1.8) Let $|{\varphi }_{n}\rangle =\frac{1}{\sqrt{N}}{\sum }_{i=0}^{N-1}|i{\rangle }_{h}|f(i,{x}_{1})\oplus f(i,{x}_{2})\oplus \cdot \,\cdot \,\cdot \oplus f(i,{x}_{n})\rangle$. Alice prepares another auxiliary state |0〉, and then applies an oracle operator *U*_*Alice*_ to $|\varphi {\rangle }_{n}\otimes |0\rangle$, where the oracle operator *U*_*Alice*_ is defined by

8 $${f}_{1}(i,{x}_{1},\ldots ,{x}_{n})=f(i,{x}_{1})\oplus f(i,{x}_{2})\oplus \cdots \oplus f(i,{x}_{n}),$$

9 $${U}_{Alice}:\frac{1}{\sqrt{N}}{\sum }_{i=0}^{N-1}|i{\rangle }_{h}|{f}_{1}(i,{x}_{1},\ldots ,{x}_{n})\rangle \otimes |0\rangle \to \frac{1}{\sqrt{N}}{\sum }_{i=0}^{N-1}|i{\rangle }_{h}|{f}_{1}(i,{x}_{1},\ldots ,{x}_{n})\rangle |0\oplus {f}_{2}(i,x)\rangle ,$$

with

10 $${f}_{2}(i,x)=\{\begin{array}{ll}1 & if\,{f}_{1}(i,{x}_{1},\ldots ,{x}_{n})=1\,and\,i\ge x\\ 0 & else\end{array}.$$

Let $|{\varphi }_{A}\rangle =\frac{1}{\sqrt{N}}{\sum }_{i=0}^{N-1}|i\rangle |{f}_{1}(i,{x}_{1},\ldots ,{x}_{n})\rangle |{f}_{2}(i,x)\rangle$. Please note that the subscript *h* is omitted in |*ϕ*_*A*_〉, because all qubits are held by Alice at this moment. Clearly,

11 $$\begin{array}{rcl}|{\varphi }_{A}\rangle  & = & \frac{1}{\sqrt{N}}[{\sum }_{i\notin \{{x}_{1},{x}_{2},\ldots {x}_{n}\}}|i\rangle |0\rangle |0\rangle +{\sum }_{j\in \{{x}_{1},{x}_{2},\ldots {x}_{n}\}\wedge j < x}|j\rangle |1\rangle |0\rangle \\  &  & +{\sum }_{j\in \{{x}_{1},{x}_{2},\ldots {x}_{n}\}\wedge j\ge x}|j\rangle |1\rangle |1\rangle ]\end{array}.$$

(1.9) Alice applies the Grover’s search algorithm^36^ to |*ϕ*_*A*_〉 for finding a marked state |*j*〉|1〉|1〉, which implies *j* ∈ {*x*_1_, *x*_2_…, *x*_*n*_} and *j* ≥ *x* (i.e., finding a bid *x*_*i*_ greater than or equal to *x*). Alice makes a measurement on the first register. Let the result of the measurement be *y*. If *y* > *x* and satisfy |*y*〉|1〉|1〉), then replace *x* with *y*.

The test procedure: (2.1) Alice first prepares a quantum state $|\psi {\rangle }_{h}=\frac{|0{\rangle }_{h}+{|i\rangle }_{h}}{\sqrt{2}}$, where *i* ∉ {*x*_1_, *x*_2_…, *x*_*n*_} (Note. *i* may be selected by Alice’s experience and the valuation price, e.g., *i* could be a large enough number in ${Z}_{N}^{\ast }$), and another quantum basis state |0〉_*t*_. Similarly, Alice further performs log*N* CNOT gate operators on the product state |*ψ*〉_*h*_|0〉_*t*_ to generate an entangled state $|{\psi }_{0}\rangle =\frac{{|0\rangle }_{h}{|0\rangle }_{t}+|i{\rangle }_{h}{|i\rangle }_{t}}{\sqrt{2}}$. Here the subscript *h* and *t* denote two registers, where the register *h* will stay at home and the register *t* will be transmitted through the quantum channel. Then Alice sends the register t to the first bidder *Bob*_1_ through the quantum channel.

(2.2) All bidders cannot distinguish the quantum states from the normal procedure and the test procedure, so they continue to execute the same oracle operators as the normal procedure (i.e., (1.2–1.5)) to mark their respective bids in the transmitted quantum state |*ψ*_*i*_〉. However, *i* ∉ {*x*_1_, *x*_2_…, *x*_*n*_}, so $|{\psi }_{n}\rangle =\frac{{|0\rangle }_{h}{|0\rangle }_{t}+|i{\rangle }_{h}|i{\rangle }_{t}}{\sqrt{2}}|0\rangle$. Finally, the bidder *Bob*_*n*_ sends all remaining qubits of the state |*ψ*_*n*_〉 back to the auctioneer Alice through the quantum channel.

(2.3) After receiving the state |*ψ*_*n*_〉, Alice again applies ${{\rm{CNOT}}}^{\otimes \mathrm{log}N}$ on two registers *h* and *t*, i.e., the first 2log*N* qubits of |*ψ*_*n*_〉, where each qubit of the first log*N* qubits is the control qubit and the corresponding qubit of the second log*N* qubits is the target qubit. Then Alice should get $|{\psi }_{n}^{\ast }\rangle =\frac{{|0\rangle }_{h}+|i{\rangle }_{h}}{\sqrt{2}}{|0\rangle }_{t}|0\rangle$.

(2.4) Furthermore, Alice measures the first register by a von Neumann measurement {*P*_+*i*_, *P*_−*i*_}, where *P*_+*i*_ and *P*_−*i*_ are defined by^37^,

12 $${P}_{+i}=\frac{1}{2}(|0\rangle \langle 0|+|0\rangle \langle i|+|i\rangle \langle 0|+|i\rangle \langle i|),$$

13 $${P}_{-i}=\frac{1}{2}(|0\rangle \langle 0|-|0\rangle \langle i|-|i\rangle \langle 0|+|i\rangle \langle i|).$$

Obviously, *P*_+*i*_ + *P*_−*i*_ = *I* and *P*_+*i*_*P*_−*i*_ = 0. If the measurement result is in $\frac{|0{\rangle }_{h}+|i{\rangle }_{h}}{\sqrt{2}}$, then she will further measure the latter two registers in computational basis. If three measurement results are in $\frac{|0{\rangle }_{h}+|i{\rangle }_{h}}{\sqrt{2}}$, |0〉_*t*_ and |0〉, respectively, then she will continue to execute the next step. Otherwise Alice will believe that there is at least one dishonest bidder or outsider attacker and end this auction.

**Step 3**. After executing the procedures of Step 2 (*p* + *q*) times, including the normal procedure *p* times and the test procedure *q* times, if the return result *y* is greater than or equal to her initial valuation price, Alice will announce *y*, i.e., the current highest bid (*y* ∈ {*x*_1_, *x*_2_, …, *x*_*n*_}). Otherwise Alice will open her commitment *x* (i.e., the initial valuation price) by opening the random number *r* simultaneously, declare the failure of the auction and terminate this auction. That is, there is not a bid greater than or equal to her initial valuation price, so this auction is fail. Of course, all participants may verify its truth by comparing *H*(*r* ⊕ *H*(*r* ⊕ *x*)) with the corresponding value *b* committed in Step 1.

**Step 4**. If there is a bid *x*_*j*_ greater than the current highest bid *y*, the bidder *Bob*_*j*_ will broadcast a complaint about the incorrectness of the current highest bid. Furthermore, if there is a complaint, Alice will ask for the bid of the complainer, and then she will update the current highest bid with it. But if there are two or more complaints, Alice will think there are dishonest bidders or outsider attackers and accordingly terminate this auction.

**Step 5**. Furthermore, if each bidder does not further receive any complaint, then he will believe that the current highest bid is highest. Suppose *y* = *x*_*k*_, i.e., the bidder *Bob*_*k*_ should be the winner of the auction. Finally, in order to win the auction successfully, the bidder *Bob*_*k*_ must publish his random number *r*_*k*_ and his bid *x*_*k*_, i.e., open his commitment. All participants will compute *H*(*r*_*k*_ ⊕ *H*(*r*_*k*_ ⊕ *x*_*k*_)) and verify its authenticity by comparing it with the corresponding value *b*_*k*_ committed in Step 1. In addition, Alice also needs to open her commitment *x* and accepts the verification of all bidders. If there is no error, the auctioneer Alice and all bidders will believe the auction is fair.

### Analysis

#### Correctness

Our PQSA scheme is based on Grover’s search algorithm, which can find a solution with a high probability^1,36^. Assume the failure probability of Grover’s search algorithm is $\frac{1}{\delta }$, where *δ* ≥ *e* (Note. *e* is the Euler’s constant, which is the base of natural logarithms (approximately 2.7183)). Let *E*(*N*, *t*) be the expectation value of the number of iterations (i.e., the number of repeating Grover’s search algorithm in Step 2) for finding the highest bid of *N* items in which *t* items are marked^38^. Then we write a recurrence equation for *E*(*N*, *t*) as:

14 $$E(N,t)=\frac{1}{t}[E(N,t-1)+\ldots +E(N,1)]+1.$$

So we get

15 $$tE(N,t)={\sum }_{i=1}^{t-1}E(N,i)+t,$$

16 $$(t-1)E(N,t-1)={\sum }_{i=1}^{t-2}E(N,i)+(t-1).$$

Subtracting Eqs (16) from (15) and rearranging, we get

17 $$E(N,t)=E(N,t-1)+\frac{1}{t}.$$

Writing the same equation for (*t* − 1), …, 2 and adding all of them, we get,

18 $$E(N,t)=E(N,1)+\frac{1}{2}+\frac{1}{3}+\cdots +\frac{1}{t}.$$

Obviously, *E*(*N*, 1) = 1. That is, there is only one marked item in the general state of *N* items, so it only needs to execute Grover’s search algorithm once to get the highest bid with the high probability of $1-\frac{1}{\delta }$. Furthermore, it will give,

19 $$E(N,t)=1+\frac{1}{2}+\frac{1}{3}+\cdots +\frac{1}{t}.$$

From Eq. (19) we can get,

20 $$E(N,t)\le {\int }_{1}^{t}\frac{1}{t}dt=\,\mathrm{ln}\,t.$$

In our PQSA scheme, there are at most *n* marked item, i.e., all bids are greater than the initial valuation price. So an upper bound is achieved for *t* = *n*, when we get,

21 $$E(N,n)\le lnn.$$

Therefore, we can repeat Grover’s search algorithm to obtain the highest bid with a probability of $1-{(\frac{1}{\delta })}^{\mathrm{ln}n}$ after ln*n* repetitions of this algorithm. That is, the failure probability *ε* of Step 2 to obtain the highest bid is ${(\frac{1}{\delta })}^{\mathrm{ln}n}$. When *δ* ≥ *e*, we can get

22 $$\varepsilon ={(\frac{1}{\delta })}^{\mathrm{ln}n}\le {(\frac{1}{e})}^{\mathrm{ln}n}\le \frac{1}{n}.$$

The failure probability of $\frac{1}{n}$ is very small, so we only tolerate a complaint in Step 4. Therefore, if all participants honestly execute the procedures, our PQSA scheme is correct.

In above analysis, we assume that Grover’s search algorithm has some probability of failure, i.e., the probability of finding the marked item is not exactly 1. Furthermore, Long^39^ presented a modified version of Grover’s search algorithm that searches a marked state with full successful rate. So, if we use Long’s algorithm in our proposed protocol, it can get the better result theoretically.

#### Security

First, we analysis the proposed scheme can resist all kinds of outsider attacks. For an outsider attacker, he can intercept the transmitted messages, including classical messages and quantum messages. If the outsider attacker wants to get *x*_*i*_ from $H({r}_{i}\oplus H({r}_{i}\oplus {x}_{i}))$ without *r*_*i*_, it is equivalent to break Hash function. At present, there is still not efficient method to break secure Hash function (e.g., SHA-1, SHA-2) by quantum computers or quantum algorithms. So, in the following we main analysis the possible attack to the transmitted quantum messages.

Firstly, the outsider attacker may perform an intercept-and-resend attack, i.e., he can intercept the transmitted quantum messages, and resend a fake quantum messages back to Alice. For example, the attacker intercepts the partial qubits of the state $|{\psi }_{n}\rangle =\frac{1}{\sqrt{N}}[{\sum }_{i\notin \{{x}_{1},{x}_{2},\ldots {x}_{n}\}}|i{\rangle }_{h}|i{\rangle }_{t}|0\rangle +{\sum }_{j\in \{{x}_{1},{x}_{2},\ldots {x}_{n}\}}|j{\rangle }_{h}|j{\rangle }_{t}|1\rangle ]$ in the normal model. Clearly, the state |*ψ*_*n*_〉 held by Alice and the attacker is an entangled state, where the reduced density matrixes of the subsystem held by them are $\frac{1}{N}{\sum }_{i=0}^{N-1}|i\rangle \langle i|$ and $\frac{1}{N}[{\sum }_{i\notin \{{x}_{1},{x}_{2},\ldots {x}_{n}\}}|i,0\rangle \langle i,0|+{\sum }_{j\in \{{x}_{1},{x}_{2},\ldots {x}_{n}\}}|j,1\rangle \langle j,1|]$, respectively. Though the reduced density matrix held by the attacker hides all private bids, the attacker cannot extract all by the principle of quantum mechanics. That is, even if the attacker measures his intercepted subsystem, he cannot get all private bids (i.e., all marked items). In fact, he can get at most one bid (i.e., one marked item) with a low probability because *n* ≪ *N*, and the bid does not reveal any identity of the bidder. However, if the attacker intercepts the partial qubits of the state $|{\psi }_{n}\rangle =\frac{{|0\rangle }_{h}|0{\rangle }_{t}+|i{\rangle }_{h}{|i\rangle }_{t}}{\sqrt{2}}|0\rangle$ in the test model, then the reduced density matrix of the subsystem held by himself is $\frac{|0,0\rangle \langle 0,0\,|+|i,0\rangle \langle i,0|}{2}$, which is independent of all bids. That is, the intercepted subsystem cannot contain any private information about any private bid.

However, the attacker cannot distinguish the transmitted quantum states from the normal model and the test model. So, if the attacker measures his intercepted subsystem to get a bid, then he will be found later by Alice with great risk. For example, if the attacker measures the state $|{\psi }_{n}\rangle =\frac{|0{\rangle }_{h}{|0\rangle }_{t}+{|i\rangle }_{h}|i{\rangle }_{t}}{\sqrt{2}}|0\rangle$ of the test model in the computation basis, the state |*ψ*_*n*_〉 will be collapsed into |0〉_*h*_|0〉_*t*_|0〉 or |*i*〉_*h*_|*i*〉_*t*_|0〉 with the probability of $\frac{1}{2}$, respectively. Later, Alice performs the test procedure in (2.4) of Step 2, so she can easily find this attack.

Of course, if the attacker sends a fake quantum system back to Alice, instead of the true subsystem intercepted by him, it will be easily found by Alice in (1.7) or (2.4) of Step 2. Therefore, our scheme can resist the intercept-and-resend attack.

Secondly, we analyze a more complicated attack, that is, the outsider attacker performs an entangle-and-measure attack that he first prepares an ancillary quantum system and further entangles his ancillary quantum system and the intercepted subsystem by a local unitary operator, and afterward he can measure the ancillary quantum system to get the partial information about the private bids. The attacker’s dishonest action can be described by a local unitary operator $\tilde{U}$, which is simply defined by,

23 $$\tilde{U}|j\rangle |0\rangle =\sqrt{{\eta }_{j}}|j\rangle |\xi (j)\rangle +\sqrt{1-{\eta }_{j}}|V(j)\rangle ,$$

where |*V*(*j*)〉 is a vector orthogonal to |*j*〉|*ξ*(*j*)〉, i.e.,

24 $$\langle j|\langle \xi (j)|V(j)\rangle =0$$

In order to completely pass the honest test (see (1.7) or (2.4) of Step 2), it can easily deduce that *η*_*j*_ = 1. That is, the whole quantum system sent back to Alice in the normal model should be in the following state after performing the operator $\tilde{U}$:

25 $$\begin{array}{rcl}\tilde{U}|{\psi }_{n}\rangle |0\rangle  & = & \tilde{U}\frac{1}{\sqrt{N}}[\sum _{i\notin \{{x}_{1},{x}_{2},\ldots {x}_{n}\}}|i{\rangle }_{h}|i{\rangle }_{t}|0\rangle +\sum _{j\in \{{x}_{1},{x}_{2},\ldots {x}_{n}\}}|j{\rangle }_{h}|j{\rangle }_{t}|1\rangle ]|0\rangle \\  & = & \frac{1}{\sqrt{N}}[{\sum }_{i\notin \{{x}_{1},{x}_{2},\ldots {x}_{n}\}}|i{\rangle }_{h}|i{\rangle }_{t}|0\rangle |\xi (i,0)\rangle +{\sum }_{j\in \{{x}_{1},{x}_{2},\ldots {x}_{n}\}}|j{\rangle }_{h}|j{\rangle }_{t}|1\rangle |\xi (j,1)\rangle .\end{array}$$

After successfully passing the honest test, the state of the whole quantum system is in,

26 $$\frac{1}{\sqrt{N}}[{\sum }_{i\notin \{{x}_{1},{x}_{2},\ldots {x}_{n}\}}|i{\rangle }_{h}|0\rangle |\xi (i,0)\rangle +{\sum }_{j\in \{{x}_{1},{x}_{2},\ldots {x}_{n}\}}|j{\rangle }_{h}|1\rangle |\xi (j,1)\rangle .$$

After performing *U*_*Alice*_ in (1.8) of Step 2, the state of the quantum system becomes,

27 $$\frac{1}{\sqrt{N}}[{\sum }_{i\notin \{{x}_{1},{x}_{2},\ldots {x}_{n}\}}|i{\rangle }_{h}|0\rangle |0\rangle |\xi (i,0)\rangle +{\sum }_{j\in \{{x}_{1},{x}_{2},\ldots {x}_{n}\}}|j{\rangle }_{h}|1\rangle |{f}_{2}(j,x)|\xi (j,1)\rangle .$$

At this moment, if the attacker measures his ancillary quantum system, then he will get *ξ*(*i*, 0) with a higher probability or *ξ*(*j*, 1) with a lower probability, because *n* ≪ *N* actually, where the latter includes a bid. However, if Alice further executes Grover’s search algorithm to find a marked state $|j\rangle |1\rangle |1\rangle |\xi (j,1)\rangle$, then the attacker will get *ξ*(*j*, 1) with a high probability. Now, he can get a bid, but he cannot distinguish his identity.

However, our scheme still has another model, i.e., the test model. If the attacker performs the entangle-and-measure attack in the test model, the whole quantum system sent back to Alice should be in the following state after performing the operator $\tilde{U}$:

28 $$\begin{array}{rcl}\tilde{U}|{\psi }_{n}\rangle  & = & \tilde{U}\frac{{|0\rangle }_{h}|0{\rangle }_{t}|0\rangle +{|i\rangle }_{h}{|i\rangle }_{t}|0\rangle }{\sqrt{2}}|0\rangle \\  & = & \frac{{|0\rangle }_{h}|0{\rangle }_{t}|0\rangle |\xi (0,0)\rangle +{|i\rangle }_{h}{|i\rangle }_{t}|0\rangle |\xi (i,0)\rangle .}{\sqrt{2}}\end{array}$$

After Alice executes the procedure of (2.3) in Step 2, the quantum system will become $|{\psi }_{n}^{\ast }\rangle =\frac{|0{\rangle }_{h}|0{\rangle }_{t}|0\rangle |\xi (0,0)\rangle +{|i\rangle }_{h}{|0\rangle }_{t}|0\rangle |\xi (i,0)\rangle }{\sqrt{2}}$. At this moment, if Alice continues to execute the test procedure of (2.4), i.e., she performs a von Neumann measurement {*P*_+*i*_, *P*_−*i*_} on the first register, then she will get the following results,

29 $${p}_{+i}=\langle {\psi }_{n}^{\ast }|{P}_{+i}\otimes I\otimes I\otimes I|{\psi }_{n}^{\ast }\rangle =\frac{1}{2},$$

30 $${p}_{-i}=\langle {\psi }_{n}^{\ast }|{P}_{-i}\otimes I\otimes I\otimes I|{\psi }_{n}^{\ast }\rangle =\frac{1}{2},$$

31 $$\frac{{P}_{+i}\otimes I\otimes I\otimes I|{\psi }_{n}^{\ast }\rangle }{\sqrt{{p}_{+i}}}=\frac{|0{\rangle }_{h}+|i{\rangle }_{h}}{\sqrt{2}}\otimes {|0\rangle }_{t}\otimes |0\rangle \otimes \frac{|\xi (0,0)\rangle +|\xi (i,0)\rangle }{\sqrt{2}},$$

32 $$\frac{{P}_{-i}\otimes I\otimes I\otimes I|{\psi }_{n}^{\ast }\rangle }{\sqrt{{p}_{-i}}}=\frac{|0{\rangle }_{h}-|i{\rangle }_{h}}{\sqrt{2}}\otimes {|0}_{t}\rangle \otimes |0\rangle \otimes \frac{|\xi (0,0)\rangle -|\xi (i,0)\rangle }{\sqrt{2}}.$$

That is, she will get $\frac{|0{\rangle }_{h}+|i{\rangle }_{h}}{\sqrt{2}}$ or $\frac{|0{\rangle }_{h}-|i{\rangle }_{h}}{\sqrt{2}}$ with the probability of $\frac{1}{2}$, respectively. Obviously, Alice will detect the attack with the probability of $\frac{1}{2}$.

Finally, we consider that the attacker tries to add some false marked items in the returned state |*ψ*_*n*_〉 by the oracle operators to manipulate the auction. On the one hand, if the false marked items are smaller than the highest bid, it will not affect the correctness of the auction; On the other hand, if a certain false marked item is greater than the highest bid, it will be easily found because no bidder claims the false bid. Even if a collusion bidder claims the false bid, obviously he will not successfully pass the public verification.

In a word, no matter which attack the outsider attacker performs, he cannot get any private information without risking Alice’s detection, and cannot manipulate the auction yet. That is, our scheme can resist the outsider attacks.

In addition, by the system model defined in the section of 3.1, PQSA should meets five secure and privacy requirements. In the following section, we will prove that our proposed PQSA scheme can meet all these secure and privacy requirements.

(1) **The auctioneer’s privacy**: From the scheme proposed above, we can easily see that the transmitted quantum messages do not include any information about Alice’s initial valuation price *x*. In addition, among all quantum oracle operators utilized by our proposed scheme, it is only the oracle operator *U*_*Alice*_ concerning *x*. However, *U*_*Alice*_ only is performed in Alice’s registers, and these quantum states transferred by the operator *U*_*Alice*_ will be measured timely by Alice. So, if a dishonest bidder (or an outsider attacker) wants to steal Alice’s private information, he can only perform the entangle-and-measure attack. However, we have analyzed the infeasibility of this attack above, because he cannot yet discern the normal model and the test model. If he performs the entangle-and-measure attack in the test model, his dishonesty will be found by Alice with the probability of $\frac{1}{2}$.

(2) **The bidder’s privacy**: As we have analyzed above, any outsider attacker cannot get any private bid without risking the auctioneer’s detection. In fact, for a bidder, he cannot get more information from the transmitted quantum messages than the outsider. If a dishonest bidder performs an attack, no matter concerned with measurement or entanglement, similarly, he will risk to be found later by the auctioneer. In short, no one can get the private bid of the bidder without risking the auctioneer’s detection.

(3) **Anonymity**: By the proposed scheme, each bidder marks his bid in the transmitted quantum state |*ψ*_*i*_〉. However, each bidder marks his bid in an anonymous way, i.e., the marked item in |*ψ*_*i*_〉 does not leave any identity.

For a dishonest bidder, e.g., *Bob*_2_, if he wants to get the specific bid of *Bob*_1_ when receiving |*ψ*_1_〉, he can perform Grover’s search algorithm to find |*x*_1_〉_*t*_|1〉 because *Bob*_2_ knows that there is only one marked item (i.e., *x*_1_) in |*ψ*_1_〉. However, if Alice selects the test model in Step 2, she can easily find this dishonesty because the final measurement result will be |0〉_*h*_ or |*i*〉_*h*_, instead of $\frac{|0{\rangle }_{h}+|i{\rangle }_{h}}{\sqrt{2}}$. That is, the dishonest bidder *Bob*_2_ cannot get the bid of the first bidder *Bob*_1_ without risking Alice’s detection. In addition, after performing Grover’s search algorithm, if *Bob*_2_ directly sends a fake state to the next bidder, not |*x*_1_〉_*t*_|1〉, obviously it will be easily found by Alice in (1.7) or (2.4) of Step 2.

As for the other bidder *Bob*_*i*_, even if he performs the similar attack to get |*x*_1_〉_*t*_|1〉 by Grover’s search algorithm, he still cannot get the specific identity of *x*_*j*_ because of *j* ∈ {1, 2, …, *i* − 1}. Even if multiple bidders collude to perform this attack, it will be found later by Alice with the probability of $\frac{q}{p+q}$. In addition, this attack also brings a risk of the failure of the auction, because our proposed scheme only permits at most one complaint when announcing the highest bid.

At present, we only assume that there is a circle quantum channel among the auctioneer and all bidders in our PQAS model. For the current technical conditions, obviously this model is more feasible. In fact, if there is a quantum channel between any two parties, the quantum messages can be transmitted in a random order, i.e., from *Bob*_*i*_ to random *Bob*_*j*_, not *Bob*_*i*+1_, such that it can provide the perfect anonymity of the bids.

For the auctioneer Alice, she can receive the returned state |*ψ*_*n*_〉, in which all bids have be marked in an anonymous way. Furthermore, she can get a marked item |*y*〉|1〉|1〉 by Grover’s search algorithm, but she cannot know *y* belongs to who because of *y* ∈ {1, 2, …, *n*}.

Therefore, our proposed scheme can ensure that the bidder’s bid is anonymous for all participants, including the auctioneer.

(4) **Public verifiability**: On the one hand, when the highest bid *x*_*k*_ is announced publicly, it needs to accept the comparisons of all other bidders to decide whether it is greater than their respective bids. On the other hand, to further win the auction successfully, the highest bidder *Bob*_*k*_ requires to open his commitment *x*_*k*_ to accept the verifications of the authenticity of the bid *x*_*k*_. As you know, there is not a perfect secure quantum bit commitment based on the No-Go Theorem^40–42^. So we utilizes a practical and efficient classical bit string commitment, in which it can not get *x*_*k*_ only from $H({r}_{k}\oplus H({r}_{k}\oplus {x}_{k}))$ without *r*_*k*_, unless cracking the secure hash function, e.g., SHA-1, SHA-2. By the opening information *r*_*k*_, anyone can verify the authenticity of the winning bid *x*_*k*_. Even if the auctioneer wants to help a malicious bidder *Bob*_*j*_ to win this auction, but they cannot revise the hash value $H({r}_{j}\oplus H({r}_{j}\oplus {x}_{j}))$, which was published in advance, so the fake bid ${r}_{j}^{\ast }$ (implying ${r}_{j}^{\ast } > {r}_{k}$) cannot pass the verification finally. That is, this attribute can defend the collusion attack between the malicious bidder and the dishonest auctioneer. In fact, bit string commitments ensures that the initial valuation price and all bids can not changed during the whole auction, otherwise the cheating will be found easily.

(5) **Fairness**: Since all bidders and the auctioneer need to commit their bids and the valuation price at the beginning of the auction, and the successfully winning bid needs to be verified publicly by all participants finally, no one can manipulate the auction, even for the auctioneer. That is, the auctioneer cannot help a malicious bidder to win the auction illegally without being found by other bidders. Therefore, our proposed scheme can guarantee the fairness of the auction.

We have analyzed the security of proposed scheme in ideal settings. However, in practical settings, there may be some faults (e.g., noise and error) in the quantum channels and quantum measurements. In order to ensure its security in practical settings, one can use the fault tolerant technologies, such as decoherence-free states and error-correcting code. In addition, we can use classical authenticated channels and quantum authenticated channels to ensure the correctness of distributing messages.

#### Performance

The proposed scheme is mainly based on Grover’s search algorithm. By the previous analysis, the number of iterations (i.e., the number of repeating Grover’s search algorithm in Step 2) for finding the highest bid is less than or equal to ln*n*, which is its upper bound, so both the computational complexity and the communicational complexity are *O*(ln*n*), i.e., to execute *O*(ln*n*) Grover’s search algorithms and to distribute *O*(ln*n*) quantum messages. To complete the task, any classical scheme needs to distribute *O*(*n*) messages in theory, where each message gets a bid in an anonymous way, and then finds the highest bid by comparing *O*(*n*) times. Obviously, our proposed quantum scheme gets the lower communicational complexity than any classical scheme.

In addition, to make our scheme work, the key step is to construct the efficient circuits implementing the oracle operators. In our scheme, we define two kinds of oracle operators to mark items in a general state. Similarly, using the techniques of reversible computation^1^, we can construct a classical reversible circuit which takes (*x*, *y*) - representing an input register initially set to *x* and a one bit output register initially set to *y* - to (*x*, *y* ⊕ *f*(*x*)), by modifying the usual (irreversible) classical circuit for doing the classical function *f*(*x*).

At present, Grover’s search algorithm and its variants have been implemented by the newest reports^43–45^, especially in IBM quantum cloud^46^. So, with the rapid development of quantum computing and quantum information processing, we believe that our proposed PQSA scheme is feasible in the near future.

## Conclusions

In this paper, we define a new privacy-preserving quantum sealed-bid auction model, and further present a novel privacy-preserving quantum sealed-bid auction scheme based on Grover’s search algorithm. The proposed scheme not only guarantees the correctness and fairness of the auction, but also ensures the privacy and anonymity of the bidders, even for the auctioneer. Compared with the current existing quantum sealed-bid auction, our proposed scheme can provide stronger privacy protections, which are urgently requirements in modern network society. So the proposed scheme has wider popularization and application prospects.

In addition, we actually give an efficient quantum approach to privately find the optimal solution under the constraint conditions among multiple distributed participants, which can also be generalized into other secure applications, e.g., an election satisfying more than half of votes.

## Data Availability

Data sharing is not applicable as no datasets were used during the current study.

## References

[1] Nielsen, M. A. & Chuang, I. L. Quantum Computation and Quantum Information: 10th Anniversary Edition, (Cambridge University Press, Cambridge, 2011).

[2] Long GL, Liu XS (2002). Theoretically efficient high-capacity quantum key distribution scheme. Phys. Rev. A.

[3] Bennett CH (1993). Teleporting an Unknown Quantum State via Dual Classical and EPR Channels. Phys. Rev. Lett..

[4] Cai XD (2015). Entanglement-based machine learning on a quantum computer. Phys. Rev. Lett..

[5] Sheng YB, Zhou L (2017). Distributed secure quantum machine learning. Sci. Bull..

[6] Bennett, C. H. & Brassard, G. Quantum Cryptography: Public Key Distribution and Coin Tossing. In: *Proc. IEEE International Conference on Computers, Systems, and Signal Processing*, pp.175–179 (1984).

[7] Hillery M, Bužek V, Berthiaume A (1999). Quantum Secret Sharing. Phys. Rev. A.

[8] Deng FG, Long GL, Liu XS (2003). Two-step quantum direct communication protocol using the Einstein-Podolsky-Rosen pair block. Phys. Rev. A.

[9] Zhang W (2017). Quantum Secure Direct Communication with Quantum Memory. Phys. Rev. Lett..

[10] Chen SS, Zhou L, Zhong W, Sheng YB (2017). Three-step three-party quantum secure direct. Sci. China-Phys. Mech. Astron..

[11] Boykin PO, Roychowdhury V (2003). Optimal encryption of quantum bits. Phys. Rev. A.

[12] Zeng G, Keitel CH (2002). Arbitrated quantum-signature scheme. Phys. Rev. A.

[13] Wang TY (2015). Security of quantum digital signatures for classical messages. Sci. Rep..

[14] Wang TY, Ma JF, Cai XQ (2017). The postprocessing of quantum digital signatures. Quantum Inf. Process..

[15] Shi RH (2016). Quantum private set intersection cardinality and its application to anonymous authentication. Inf. Sci..

[16] Wang TY, Wen QY, Zhu FC (2009). Secure authentication of classical messages with decoherence-free states. Opt. Commun..

[17] Fitzsimons JF (2017). Private quantum computation: an introduction to blind quantum computing and related protocols. NPJ Quantum Inf..

[18] Sheng YB, Zhou L (2018). Blind quantum computation with a noise channel. Phys. Rev. A.

[19] Hillery M (2006). Quantum voting and privacy protection: first steps. Int. Soc. Opt. Eng.

[20] Naseri M (2009). Secure quantum sealed-bid auction. Opt. Commun..

[21] Zhang JZ, Yang YY, Xie SC (2017). A Third-Party E-Payment Protocol Based on Quantum Group Blind Signature. Int J Theor Phys.

[22] Qin SJ (2009). Cryptanalysis and improvement of a secure quantum sealed-bid auction. Opt. Commun..

[23] Yang YG, Naseri M, Wen QY (2009). Improved secure quantum sealed-bid auction. Opt. Commun..

[24] Liu YM (2009). Revisiting Naseri’s secure quantum sealed-bid auction. Int. J. Quantum Inf..

[25] Zheng Y, Zhao Z (2009). Comment on: “Secure quantum sealed-bid auction” [Opt. Comm. 282 (2009) 1939]. Opt. Commun..

[26] Zhao Z, Naseri M, Zheng Y (2010). Secure quantum sealed-bid auction with post-confirmation. Opt. Commun..

[27] Xu GA (2011). Cryptanalysis and improvement of the secure quantum sealed-bid auction with postconfirmation. Int. J. Quantum Inf..

[28] He LB (2012). Cryptanalysis and melioration of secure quantum sealed-bid auction with post-confirmation. Quantum Inf. Process..

[29] Wang QL, Zhang WW, Su Q (2014). Revisiting “The loophole of the improved secure quantum sealed-bid auction with post-confirmation and solution”. Int. J. Theor. Phys..

[30] Zhang YW (2010). Quantum secure direct communication and quantum sealed-bid auction with EPR pairs. Commun. Theor. Phys..

[31] Wen JL (2014). Attacks and improvement of quantum sealed-bid auction with EPR pairs. Commun. Theor. Phys..

[32] Wang JT (2015). A new quantum sealed-bid auction protocol with secret order in post-confirmation. Quantum Inf. Process..

[33] Liu WJ (2016). Multiparty quantum sealed-bid auction using single photons as message carrier. Quantum Inf. Process..

[34] Zhang R (2018). An economic and feasible Quantum Sealed-bid Auction protocol. Quantum Inf. Process..

[35] Shi RH (2016). Secure Multiparty Quantum Computation for Summation and Multiplication. Sci. Rep..

[36] Grover, L. K. A fast quantum mechanical algorithm for database search. In: *Proc. 28th Annual ACM Symposium on Theory of Computing, ACM*, pp.212–219 (1996).

[37] Shi RH (2016). Comment on “Secure quantum private information retrieval using phase-encoded queries”. Phys. Rev. A.

[38] Ahuja, A. & Kapoor, S. A Quantum Algorithm for finding the Maximum. arXiv:quant-ph/9911082v1.

[39] Long GL (2001). Grover algorithm with zero theoretical failure rate. Phys. Rev. A.

[40] Lo HK (1997). Insecurity of quantum secure computations. Phys. Rev. A.

[41] Colbeck R (2007). The impossibility of secure two-party classical computation. Phys. Rev. A.

[42] Buhrman H, Christandl M, Schaffner C (2012). Complete Insecurity of Quantum Protocols for Classical Two-Party Computation. Phys. Rev. Lett..

[43] Chuang IL, Gershenfeld N, Kubinec M (1998). Experimental implementation of fast quantum searching. Phys. Rev. Lett..

[44] Brickman K-A (2005). Implementation of Grover’s quantum search algorithm in a scalable system. Phys. Rev. A.

[45] Figgatt C (2017). Complete 3-Qubit Grover search on a programmable quantum computer. Nat. Commun..

[46] Majumder, A., Mohapatra, S. & Kumar, A. Experimental Realization of Secure Multiparty Quantum Summation Using Five-Qubit IBM Quantum Computer on Cloud. arXiv:1707.07460v3 (2017).

